# Antimicrobial Photodynamic Therapy and Dental Plaque: A Systematic Review of the Literature

**DOI:** 10.1155/2014/824538

**Published:** 2014-10-14

**Authors:** G. C. Santin, D. S. B. Oliveira, R. Galo, M. C. Borsatto, S. A. M. Corona

**Affiliations:** ^1^Department of Pediatric Clinics, Preventive and Community Dentistry, Dental School of Ribeirão Preto, University of São Paulo, Ribeirão Preto, SP, Brazil; ^2^Department of Prosthesis and Dental Materials, Dental School of Araraquara, São Paulo State University, Araraquara, SP, Brazil; ^3^Department of Restorative Dentistry, Dental School of Ribeirão Preto, University of São Paulo, Avenida do Café, s/n, Monte Alegre, 14040-904 Ribeirão Preto, SP, Brazil

## Abstract

*Background*. The aim of this study was to perform a systematic review of the literature on the efficacy of antimicrobial photodynamic therapy (PDTa) on cariogenic dental biofilm. *Types of Studies Reviewed*. Studies *in vivo*, *in vitro*, and *in situ* were included. Articles that did not address PDTa, those that did not involve cariogenic biofilm, those that used microorganisms in the plankton phase, and reviews were excluded. Data extraction and quality assessments were performed independently by two raters using a scale. *Results*. Two hundred forty articles were retrieved; only seventeen of them met the eligibility criteria and were analyzed in the present review. Considerable variability was found regarding the methodologies and application protocols for antimicrobial PDTa. Two articles reported unfavorable results. *Practical Implications*. The present systematic review does not allow drawing any concrete conclusions regarding the efficacy of antimicrobial PDTa, although this method seems to be a promising option.

## 1. Background

Dental caries has a multifactor etiology, including cariogenic microorganisms in the oral cavity. These microorganisms use a glycolytic pathway to produce acids that are capable of demineralizing tooth enamel and dentin. Some microorganisms use sucrose as substrate for the production of intracellular and extracellular polysaccharides, which are highly cariogenic [1, 2]. Moreover, a large portion of periodontopathogenic bacteria is found in dental biofilm (plaque), which underscores the considerable contribution of this substance in the development of adverse health conditions of the oral cavity.

Dental biofilm is a three-dimensional structure of bacterial communities adhered to the tooth surface [3]. Microcolonies of bacterial cells account for 15 to 20% of dental biofilm and the rest is composed of exopolysaccharides, water, proteins, salts, and the cell fragments [4, 5]. Pores or channels of water among the bacterial microcolonies serve as a primitive circulation system, allowing the passage of nutrients and other agents, which affect the distribution and movement of molecules in biofilm [3]. The constitution of biofilm protects colonizing species from adverse factors in the environment, such as defense mechanisms of the host and potentially toxic substances (lethal chemical agents and antibiotics) [4]. Moreover, slow-growing cells, which are one of the characteristics of bacteria found in deeper portions of biofilm, are less sensitive to antimicrobial agents and the ability of bacteria in the biofilm to produce neutralizing agents that protects neighboring organisms [3]. Thus, studies have described an increase in resistance to antibiotics, due to their inadequate or excessive use [6, 7], as well as difficulties concerning the access of topical agents with effectiveness against the biofilm [8].

Chlorhexidine is a cationic broad-spectrum antimicrobial agent that has been widely studied and proven effective at controlling dental biofilm [9]. This effectiveness is directly related to a property denominated substantivity, by which the molecule remains adhered to tissues and has antibacterial action for up to 12 hours [10]. However, side effects lasting for more than 14 days are associated with chlorhexidine, such as pigmentation of the teeth and mucosa, an increase in the formation of supragingival calculus, a temporary loss of the sense of taste, a burning sensation, and dry mouth [9].

Antimicrobial photodynamic therapy (PDT) has emerged as an alternative to antibiotics for the treatment of microbial infections [6]. With this method, a photosensitizing agent is activated by light at a specific wavelength that corresponds to maximum absorbance by the substance, resulting in the production of free radicals, singlet oxygen, and other reactive oxygen species, which have a toxic effect on bacterial cells, leading to cell death without causing harm to the host [6, 7, 11–13]. This minimally invasive method is effective against resistant bacteria [14], has a rapid effect on the target organisms [15, 16], and does not lead to the development of resistance mechanisms [6, 17]. Moreover, antimicrobial PDT is selective and painless and does not affect the patient's sense of taste [18].

Most oral bacteria do not absorb visible light from some type of low-power laser light. Therefore, nontoxic optical agent absorption used to be fixed at the bacterial walls, attracting to itself the laser at the moment of irradiation. It is essential to have antimicrobial action. Reactive oxygen species released by the association between the dye and the light source causes damage to various cellular structures, but primarily to DNA and cytoplasmic membrane [12], which affects differently gram-positive and gram-negative bacterias [34]. The cellular destruction depends on the association between the dye and the light source [7, 17]. The effect of the dye is influenced of the kind, dose and site application. The efficacy of light can be influenced by wavelength, power density, energy fluence and the amount of oxygen available for the combination of both (dye and light) [11].

Different types of photosensitizers and light sources under various conditions have been used for the realization of photodynamic antimicrobial therapy [13]. For its use, a photosensitizer should have photophysical, chemical, and biological characteristics appropriate [11] among which is the ability to become an active drug and provide singlet oxygen, a broad spectrum of action, and affinity for microorganisms; low affinity for host cells promotes low mutagenicity and cytotoxicity associated with low possibility of developing resistant strains of microorganisms [11]. The light source, to be adequate, must present low power situated in the visible portion of the electromagnetic spectrum and specific wavelength resonant to dye. The wavelength depends on the dose and the depth of action of the photosensitizer used [11].

However, bacteria in biofilm have been demonstrated to be less affected by PDT than those in the plankton phase [35]. While a number of authors working with different light sources and photosensitizing agents report the efficacy of PDT in controlling dental biofilm by reducing the bacteria viability [7, 13, 14, 16, 22–24, 36–38], there is a lack of scientific evidence regarding the actual effectiveness of this method. Thus, the aim of the present study was to perform a systematic review of the literature on the efficacy of antimicrobial PDT on dental biofilm.

## 2. Methods

Articles addressing the effect of antimicrobial PDT with the use of a photosensitizing agent on known cariogenic biofilm formed mainly by streptococci of the* mutans* group and/or lactobacilli were included, with no restrictions placed on the method employed or year of publication. Reviews of the literature, studies involving only bacteria in the plankton phase, and studies involving an animal model were excluded.

### 2.1. Search Strategy

Searches were made of the Pubmed, Web of Science, Scopus, Lilacs, and Cochrane Library databases in October and November 2013 as well as March 2014. A manual search of the references of each article retrieved was also performed in an attempt to find further articles that were not in the electronic databases. Each database was searched from its inception to March 2014. The search was performed by two researchers and limited to studies involving human subjects published in the English language, using the following keywords: (“Biofilms”[Mesh] OR “Dental Plaque”[Mesh]) AND (photodynamic therapy OR antimicrobial photodynamic therapy OR light therapy).

### 2.2. Data Extraction and Evaluation of Methodological Quality

**Figure 1:**
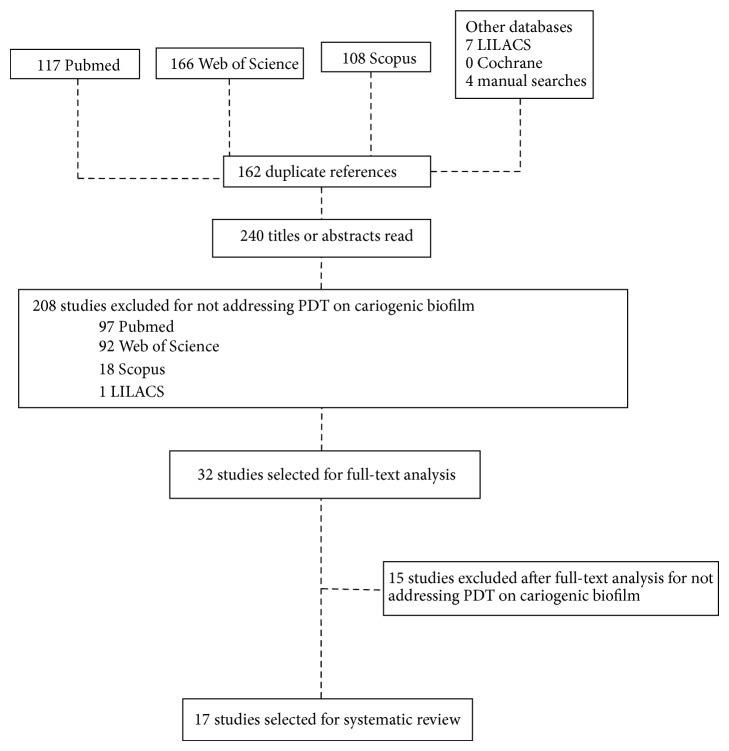
Flowchart demonstrating selection process of studies on PDT and dental biofilm.

A total of 23 articles were retrieved from the databases and four additional articles were retrieved from the manual search of the reference lists. Following the reading of the title and abstract of each article, two independent raters (GCS and DSBO) selected studies for the full-text analysis. Interexaminer agreement was 96%. Thirty-two articles were selected for the full-text analysis due to insufficient information in the abstract to support the decision regarding eligibility. Articles that did not address antimicrobial PDT, those that did not involve potentially cariogenic biofilm, those that used microorganisms in the plankton phase, and reviews of the literature were excluded. After the full-text analyses, seventeen articles were included in the present systematic review (Figure 1).

Data extraction and the evaluation of methodological quality were performed by two independent raters (GCS and DSBO). The evaluation involved the use of a chart considering the sample (sample size calculation = 1; randomization = 1), study design (*in vivo* = 3;* in situ* = 2;* in vitro* = 1), control group (present = 1; absent = 0), blinding (double-blind = 2; single-blind; absent = 0), and repetition of the experiment (yes = 1; no = 0). The maximum score was 9 points. Disagreements between the raters were discussed and resolved by consensus. The determination and critical analysis of the quality of the articles allowed suggestions for improvements in future studies.

## 3. Results

Among the total of 240 articles retrieved during the original search of the databases and references lists, seventeen were selected for the present systematic review for addressing the efficacy of antimicrobial PDT on biofilm with cariogenic potential. All seventeen articles described either* in vitro* or* in situ *studies.  Table 2 offers a summary of the findings.

Considerable variability among the articles was found regarding the photosensitizing agent. Toluidine blue was the most commonly employed. Each study used a specific light source (LED, laser, and light bulb), power, and application protocol.

Biofilm was cultivated in human saliva in three studies [14, 22, 32], natural human biofilm was used in four studies [19, 21, 27, 30], and synthesized biofilm was used in ten studies [16, 20, 23–26, 28, 29, 31, 33].

Two articles reported unfavorable results regarding the reduction of microorganisms in dental biofilm with the use of antimicrobial PDT [25, 30].

In the analysis of methodological quality, scores ranged from 1 to 5 points. The main drawbacks were related to the sample size calculation, randomization of the sample, blinding, and repetition of the experiment (Table 1).

## 4. Discussion

The analysis of the articles revealed the frequent lack of randomization of the specimens studied and failure to calculate the appropriate sample size. These data indicate possible selection bias. Moreover, divergent effects may have derived from systemic alterations in the different specimens and there is no possibility of reproducing the studies.

The majority of articles evaluated antimicrobial PDT on dental biofilm using an* in vitro* study, which is not the best design for arriving at adequate scientific evidence, although this model has led to significant advances in the study of dental biofilm [39].* In vitro* models tend to involve a small number of species of microorganisms and laboratory conditions that may not adequately reflect the physiological situation in the oral cavity [40]. Factors such as salivary flow, the capacity of antimicrobial substances to adhere to the film on the teeth or the surface of soft tissues, and the interaction of noncultivatable bacteria cannot be modeled in an* in vitro* experiment [41].

The presence of polymeric extracellular substances, composition of the cell wall, growth rate, metabolic activity, and gene expression offer natural biofilm protection from the action of antimicrobial agents [42]. Moreover, nutritional status, temperature, pH, and undereffective exposure to antimicrobial agents can enhance bacterial resistance to this type of treatment [4, 43]. As biofilm is dependent on a number of factors, the use of a synthesized biofilm may not demonstrate the same scientific evidence as natural biofilm.

Dental biofilm has an organized structure formed by different types of microorganisms, which give the substance a complex, protective trait. Thus, studies employing biofilm composed of a single genus of microorganisms [14, 16, 19, 20, 22–24, 26, 28, 29, 31] may not demonstrate the actual effect of antimicrobial PDT on dental biofilm in the oral cavity.

Although the majority of studies report favorable results with the use of antimicrobial PDT to reduce the volume of cariogenic microorganisms in the oral cavity, the articles offered a considerable variety of photosensitizing agents, light sources, application protocols, and methods for evaluating the effectiveness of the technique. This hinders the comparison of the findings, the reproducibility of events, and the determination of possible causality between the reduction in microorganisms and antimicrobial PDT. Moreover, the variations among the methods employed hamper the establishment of a possible protocol for the application of antimicrobial PDT on cariogenic biofilm.

Most of the articles included in this review used as photosensitizing dyes phenothiazine (methylene blue and toluidine blue). The physicochemical properties of the photosensitizers are important to the efficacy of photodynamic therapy. The ability of a component to absorb incident light does not mean it can act as a photosensitizer. Other requirements are important, such as having no toxic characteristics to the host cell, presenting toxicity only after activation by light, staying excited long enough to allow its interaction with neighboring molecules, producing cytotoxic species capable of causing bacterial killing time, and having high solubility in water [8, 44, 45].

In oral antimicrobial photodynamic therapy, toluidine blue and methylene blue photosensitizing agents are the most commonly used [11, 12], since they have a high degree of selectivity for damage for gram-positive and gram-negative bacterias [46–48]. What determines the selectivity of this type of dye microbial cells is the interaction between the positive charges of the dye and the negative charges of the outer surface of the microbial cell [49]. The dye methylene blue is a prototype of phenothiazine derivatives and their use is attested almost a century and its relatively low toxicity to humans [50]. However, Wood et al. 2006 [23] noted the erythrosine better efficiency when compared to methylene blue and Photofrin on* Streptococcus mutans* biofilm.

The concentration of photosensitizers is still controversial. Al-Ahmad et al. 2013 [32] using different concentrations of toluidine blue (5, 10, 25, and 50 mg/mL^−1^) found that the antimicrobial effect can be observed at lower concentrations.

The first light sources used in photodynamic therapy were conventional lamps with noncoherent, polychromatic light and a strong thermal component. With the development of lasers, which have particular characteristics, such monochromaticity, coherence, and collimation, the light source proved to be more efficient to photodynamic therapy. Diode lasers have resonant wavelength absorption band of most currently used dyes, act continuously, and are less portable and low in cost [51]. Currently, the light of a specific wavelength, sources which are most commonly applied in PDT are helium-neon (HeNe) lasers, (633 nm) gallium-aluminum-arsenide (GaAlAs) diode lasers (630–690, 830, or 906 nm), and argon lasers (488–514 nm).

In this review, the majority of included studies used HeNe laser and LED (light emitting diode). LEDs are another alternative source of laser light and differ by presenting divergent beam, low thermal component and monochromatic light [51], and low cost [16]. Additionally, LED sources are present in the dental routine and can be used in PDT without requiring the acquisition of new equipment. However, no difference regarding the efficacy of these two types of light source for photodynamic therapy was observed [16].

Considering the wavelength, blue light has been shown to be more efficient to be used in conjunction with a dye than red light [52, 53]. However, the use of different types of light sources by authors evaluated in this review shows that there is no consensus regarding the type of light and the parameters to be used permanently in photodynamic therapy to control biofilm.

Two studies reported unfavorable results regarding the effect of antimicrobial PDT on dental biofilm [25, 30]. The authors attribute these findings to an increase in resistance to this technique among bacteria in biofilm and/or multispecies biofilm [25, 30] as well as the thickness [30] and age of the biofilm [25]. Similarly, resistance to antiseptics, such as chlorhexidine, has been described [54]. Moreover, antimicrobial efficacy is believed to be dose dependent [55], which would explain the lack of a positive effect, as antimicrobial PDT was only applied once in both studies [30]. The difficulty for antimicrobial agents, photosensitizing agents, and light to penetrate the deeper layers of biofilm limits their effectiveness [25, 30]. However, studies have demonstrated that although antimicrobial PDT is threefold to fourfold less effective on thick, multispecies biofilm, antibiotics are as much as 250-fold less effective [56]. There are alternatives that can enhance the effectiveness of antimicrobial PDT, such as the selection of photosensitizing agents capable of penetrating the matrix of the biofilm, the use of photomechanical waves to optimize the penetration of the photosensitizing agent [57], and internal irradiation of the biofilm using an optic fiber [22].

Some of the included studies investigated alternatives to optimize antimicrobial photodynamic therapy when applied in biofilm. The use of the visible light in combination with water-filtered infrared-A (VIS + wIRA) [32] showed satisfactory results, while the use of ozone gasiform [25] was not effective. The use of curcumin in biofilm decreased from 95 to 99.9% of viable microorganisms, depending on the concentration of the photosensitizing agent; however, when applied to carious dentin, their effectiveness was probably reduced by the difficulty of penetration of the photosensitizing agent [33].

*Suggestions for Future Research.* Despite the number of studies on antimicrobial PDT, greater knowledge is needed regarding the effectiveness of this form of treatment. Studies with methodological standardization, randomization, an adequate sample size, reproducibility, and adequate data analysis are needed. Moreover, the effectiveness of antimicrobial PDT on multispecies biofilm under real conditions, such as in an* in situ *and* in vivo* design, is needed to gain a better understanding of the action mechanism of this treatment modality and the determination of a possible application protocol.

## 5. Conclusion

The present systematic review of the literature does not allow drawing any concrete conclusions regarding the efficacy of antimicrobial PDT due to the contradictory findings and methodological differences. Although this method seems to be a promising option for reducing the quantity of cariogenic microorganisms in dental biofilm, there is no sufficiently strong scientific evidence to support this association.

Further experimental studies with methodological standardization, the use of natural human biofilm, and an* in vivo* design are needed to gain a better understanding of the mechanisms, indications, and possible side effects of antimicrobial photodynamic therapy.

## Figures and Tables

**Table 1:** Quality scores of articles selected based on proposed evaluation scale.

Authors and year	Sample	Study design	Control group	Blinding	Repetition of experiment	Total
Wilson et al., 1995 [19]	0	1	1	0	0	2
Wilson et al., 1996 [20]	0	1	0	0	0	1
Wood et al., 1999 [21]	0	2	1	0	0	3
O'Neill et al., 2002 [22]	0	1	1	0	0	2
Zanin et al., 2005 [16]	0	1	1	0	0	2
Wood et al., 2006 [23]	0	1	1	0	1	3
Zanin et al., 2006 [24]	1	1	1	0	0	3
Müller et al., 2007 [25]	0	1	1	0	1	3
Steinberg et al., 2008 [26]	0	1	1	0	1	3
Lima et al., 2009 [27]	1	1	1	1	0	4
Schneider et al., 2012 [14]	0	1	1	0	0	2
Chen et al., 2012 [28]	0	1	1	0	0	2
Silva et al., 2012 [29]	0	1	1	0	1	3
Teixeira et al., 2012 [30]	1	2	1	1	0	5
Pereira et al., 2013 [31]	0	1	1	0	0	2
Al-Ahmad et al., 2013 [32]	0	2	1	0	1	4
Araújo et al., 2014 [33]	0	1	1	0	1	3

**Table 2:** Summary of data reported in selected articles addressing effectiveness of antimicrobial PDT on potentially cariogenic biofilm.

Authors and year of publication	Sample	Photosensitizing agent	Light source	Microorganisms/biofilm	Application protocol	Outcome
Wilson et al., 1995 [19]	Natural dental biofilm; 10 volunteers; prereduced Calgon Ringer's solution	AlPcS_2_ TB	GaAlAs laser 660 nm (11 mW) HeNe laser 632.8 nm (7.3 mW)	*Streptococci *	60 s and 180 s	HeNe/TB combination was more effective at reducing viable microorganisms

Wilson et al., 1996 [20]	Synthesized biofilm in 4 days; single species; HA discs	AlPcS_2_	GaAlAs laser 660 nm	*S. sanguinis *	0.8 J (4.1 J/cm^2^)	Reduction in viable microorganisms

Wood et al., 1999 [21]	Natural dental biofilm in 7 days; 8 volunteers; devices were attached to surface of maxillary first or second molars	PPC	Tungsten white bulb 600–700 nm (400 w; 22.5 mW/cm^2^)	Natural human dental biofilm (species not listed)	30 minutes	Reduction in viable microorganisms

O'Neill et al., 2002 [22]	Biofilm formed from human saliva from 10 volunteers; multispecies biofilms grown on discs prepared from cellulose nitrate membrane filters for 24 h	TB	HeNe laser 632 nm (35 mW)	*Streptococci *	15 minutes (31.5 J; 81.9 J/cm^2^)	Reduction in viable microorganisms

Zanin et al., 2005 [16]	Synthesized biofilm in 3, 7, and 10 days; HA discs; single species	TB	HeNe laser 632.8 nm (32 mW) LED 620–660 nm	*S. mutans *	5 (49 J/cm^2^), 15 (147 J/cm^2^) and 30 (294 J/cm^2^) minutes	LED/TB on biofilm for 3 (147 J/or 294 J/cm^2^) and 7 days (49 J/cm^2^ or 294 J/cm^2^) For 10 days, no difference between HeNe and LED

Wood et al., 2006 [23]	Synthesized biofilms in 48, 120, 168, 216, and 288 hours; steel discs; single species	ER MB Photofrin	Tungsten white light 40 W (ER—22.7 mW/cm^2^, 500–550 nm; MB and Photofrin—22.5 mW/cm^2^, 600–650 nm)	*S. mutans *	15 minutes of irradiation	Reduction in viable microorganisms Greater effectiveness with ER

Zanin et al., 2006 [24]	Synthesized biofilm in 3, 5, and 7 days; single species; bovine enamel discs	TBO	Red LED 620–660 nm (32 mW)	*S. mutans, S. sobrinus, S. sanguinis *	7 minutes of irradiation 85.7 J/cm^−2^	Reduction in viable microorganisms

Müller et al., 2007 [25]	Synthesized biofilm in 64.5 h; multispecies; bovine enamel discs	MB	Gasiform ozone and laser 665 nm (75 mW)	*S. sobrinus, S. oralis *	60 s of irradiation	No reduction in viable microorganisms

Steinberg et al., 2008 [26]	Synthesized biofilm in 24 h; single species; microplates	H_2_O_2_	Blue xenon lamp 400–500 nm	*S. mutans *	30 s of irradiation (34 J/cm^2^) 60 s (68 J/cm^2^)	Reduction in viable microorganisms

Lima et al., 2009 [27]	Natural dental biofilm for 7 days; 20 volunteers; palatal devices containing slabs of human dentin	TB	LED 620–660 nm	*Streptococci, Lactobacilli *	5 minutes of irradiation (47 J/cm^−2^) and 10 minutes (94 J/cm^−2^)	Reduction in viable microorganisms with LED/TB and LED (94 J/cm^−2^) alone

Schneider et al., 2012 [14]	Biofilm from saliva from 4 volunteers and growth in 4 hours Glass surface Single species	Phenothiazine chloride	Diode laser 660 nm (100 mW)	*S. mutans *	2 minutes of irradiation	Reduction in viable microorganisms

Chen et al., 2012 [28]	Synthesized biofilm in 24 hours Single species Stainless steel discs	Nanoparticles of ER + CS	Green LED 540 ± 5 nm (22m W/cm^2^)	*S. mutans *	ER/CS nanoparticles for 12 h followed by dose of 50 J cm^2^	Reduction in viable microorganisms

Silva et al., 2012 [29]	Synthesized biofilm in 24 hours Single species Bovine dentin discs	Photogem hematoporphyrin derivative	Biotable LED 610–650 nm	*S. mutans *	25 minutes of irradiation (75 Jcm^−2^) and 50 minutes of irradiation (150 Jcm^−2^)	Reduction in viable microorganisms

Teixeira et al., 2012 [30]	Natural dental biofilm in 7 days Acrylic palatal device with human enamel slabs 21 volunteers	TB	Red LED 620–660 nm	*S. mutans *	15 minutes of irradiation (55 J cm^−2^)	No reduction in viable microorganisms

Pereira et al., 2013 [31]	Synthesized biofilm in 48 hours Single species Acrylic resin discs	ER and RB	Blue LED 455 ± 20 nm (200 mW)	*S. mutans, S. sanguinis *	95 Jcm^−^² (36 J and for 180 s)	Reduction in viable microorganisms

Al-Ahmad et al., 2013 [32]	Culture of microorganisms from the saliva of a volunteer of 45 years old	TB	Broadband VIS + wIRA radiator with a water-filtered (580–1400 nm)	Salivary bacteria (species not listed)	220 mW cm^−2^ For 1 min	Reduction in viable microorganisms

Araújo et al., 2014 [33]	Synthesized biofilm in 7 days Single species Polystyrene 96-well plates	Curcumin	Blue LED 450 nm	*S. mutans, L. acidophilus *	5 minutes 5, 7 J/cm^2^ (19 mW/cm^2^)	Reduction in viable microorganisms

AlPcS_2_: aluminum disulfonate phthalocyanine; GaAlAs: gallium-aluminum-arsenide; HA: hydroxyapatite; HeNe: helium/neon; LED: light emitting diode; PPC: pyridinium Zn(II) phthalocyanine; TB: toluidine blue; ER: erythrosine; TB: toluidine blue; MB: methylene blue; H_2_O_2_: hydrogen peroxide; CS: chitosan; ER: erythrosine; TBO: toluidine blue; RB: rose bengal; VIS + wIRA: visible light together with water-filtered infrared-A.
